# Tunable Temperature-Sensitive Transcriptional Activation Based on Lambda Repressor

**DOI:** 10.1021/acssynbio.2c00093

**Published:** 2022-06-16

**Authors:** Lealia L. Xiong, Michael A. Garrett, Marjorie T. Buss, Julia A. Kornfield, Mikhail G. Shapiro

**Affiliations:** † Division of Engineering and Applied Sciences, California Institute of Technology, 1200 E. California Blvd., Pasadena, California 91125, United States; ‡ Division of Chemistry and Chemical Engineering, California Institute of Technology, 1200 E. California Blvd., Pasadena, California 91125, United States; § Howard Hughes Medical Institute, California Institute of Technology, 1200 E. California Blvd., Pasadena, California 91125, United States

**Keywords:** thermal control, temperature, transactivation, transcription factors, microbial synthetic biology

## Abstract

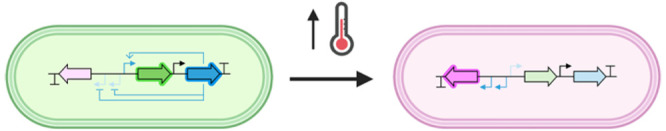

Temperature is a versatile input signal for the control of engineered cellular functions. Sharp induction of gene expression with heat has been established using bacteria- and phage-derived temperature-sensitive transcriptional repressors with tunable switching temperatures. However, few temperature-sensitive transcriptional activators have been reported that enable direct gene induction with cooling. Such activators would expand the application space for temperature control. In this technical note, we show that temperature-dependent versions of the Lambda phage repressor CI can serve as tunable cold-actuated transactivators. Natively, CI serves as both a repressor and activator of transcription. Previously, thermolabile mutants of CI, known as the TcI family, were used to repress the cognate promoters PR and PL. We hypothesized that TcI mutants can also serve as temperature-sensitive activators of transcription at CI’s natural PRM promoter, creating cold-inducible operons with a tunable response to temperature. Indeed, we demonstrate temperature-responsive activation by two variants of TcI with set points at 35.5 and 38.5 °C in *E. coli*. In addition, we show that TcI can serve as both an activator and a repressor of different genes in the same genetic circuit, leading to opposite thermal responses. Transcriptional activation by TcI expands the toolbox for control of cellular function using globally or locally applied thermal inputs.

Spatiotemporal control of engineered microbes enables patterning and localization of microbial activity in applications ranging from in vivo therapeutics to engineered living materials. Temperature can be applied globally or with spatial specificity as a deeply penetrant, noninvasive input signal.^1^ Previous work developed two families of orthogonal tunable thermal bioswitches based on bacteria- and phage-derived transcriptional repressors.^2^ These gene circuit components, along with most other currently used bacterial temperature-dependent regulators, such as heat shock factors and 5′ UTR RNA hairpins, turn on gene expression in response to increases in temperature,^3−7^ whereas few synthetic or natural cold-inducible switches have been reported.^8−11^ However, induction of gene expression based on decreases in temperature would, for example, allow for programming microbial therapeutics to self-destruct in response to leaving the body^12−14^ or engineering microbe-based living materials to activate adaptive measures beyond the native cold shock pathway^15^ in response to decreases in ambient temperature. Most existing sensors for decreases in temperature, such as the native cold shock response, have unknown tunability, narrowing the range of possible applications.^16^ Meanwhile, the inversion of hot-on bioswitches to obtain cold-on responses by adding enzymatic degradation,^8^ antirepressors,^11^ or additional repressors^2^ increases gene circuit complexity.

One of the most promising classes of thermal bioswitch repressors are mutants of CI857^17,18^ (here referred to as TcI39). This mutant of bacteriophage Lambda repressor CI has been tuned by directed evolution to transition at different set point temperatures while retaining sharp switching behavior.^2^ To date, these mutated TcI transcription factors have been applied only as repressors for hot-on gene expression, acting at cognate promoters PR and PL. However, in nature, wildtype CI also activates transcription at promoter PRM, which allows it to serve as a DNA damage-sensitive switch controlling the induction of phage Lambda from the lysogenic to the lytic phase.^19,20^ Previous interest in TcI39’s ability to activate PRM involved expression of TcI39 itself from PRM, while the main purpose of TcI39 was to regulate hot-on expression of a protein of interest from the PR promoter.^21,22^

In this technical note, we examine the ability of tunable TcI proteins to serve as cold-on transcriptional activators of specific genes of interest. We demonstrate temperature-responsive activation by two TcI variants with transition set points at 35.5 and 38.5 °C in *E. coli*. In addition, we show that a single TcI protein can act simultaneously as a temperature-responsive repressor and activator of two separate genes in a single circuit, enacting complementary thermal regulation.

Different applications of thermal control may require different temperature thresholds for gene activation. Thus, we characterized the ability to activate transcription of two previously developed mutants, TcI38 and TcI39, with bioswitch activation midpoints of 38 and 39 °C, respectively.^2^ We constructed a model gene circuit driving the expression of the mWasabi green fluorescent protein (GFP) from the PRM promoter. In its native bacteriophage Lambda, CI binds three operator sites at the bidirectional PR/PRM promoter: OR1, OR2, and OR3. CI preferentially binds OR1 and then recruits its own binding to OR2, repressing PR and activating PRM.^20^ At high concentrations, CI also binds to OR3 and represses PRM; we used a mutated OR3 to prevent this repression.^23^

In our cold-on circuit, transcription from the PRM promoter is activated by either TcI38 or TcI39, which is in turn expressed both from PRM readthrough and a weak constitutive LacI promoter (Figure 1a). For comparison, we also constructed a circuit in which wildtype CI serves as the transactivator (Figure 1b). Wildtype CI is nominally temperature independent; however, its ability to bind to operator DNA decreases gradually with increasing temperature,^24^ while its ability to activate PRM decreases below 37 °C.^25^ It was therefore important to compare TcI mutant activation profiles to wildtype CI at each temperature. Finally, we included a construct with no activator to measure the thermal profile of background gene expression at the PRM promoter (Figure 1c).

**Figure 1:**
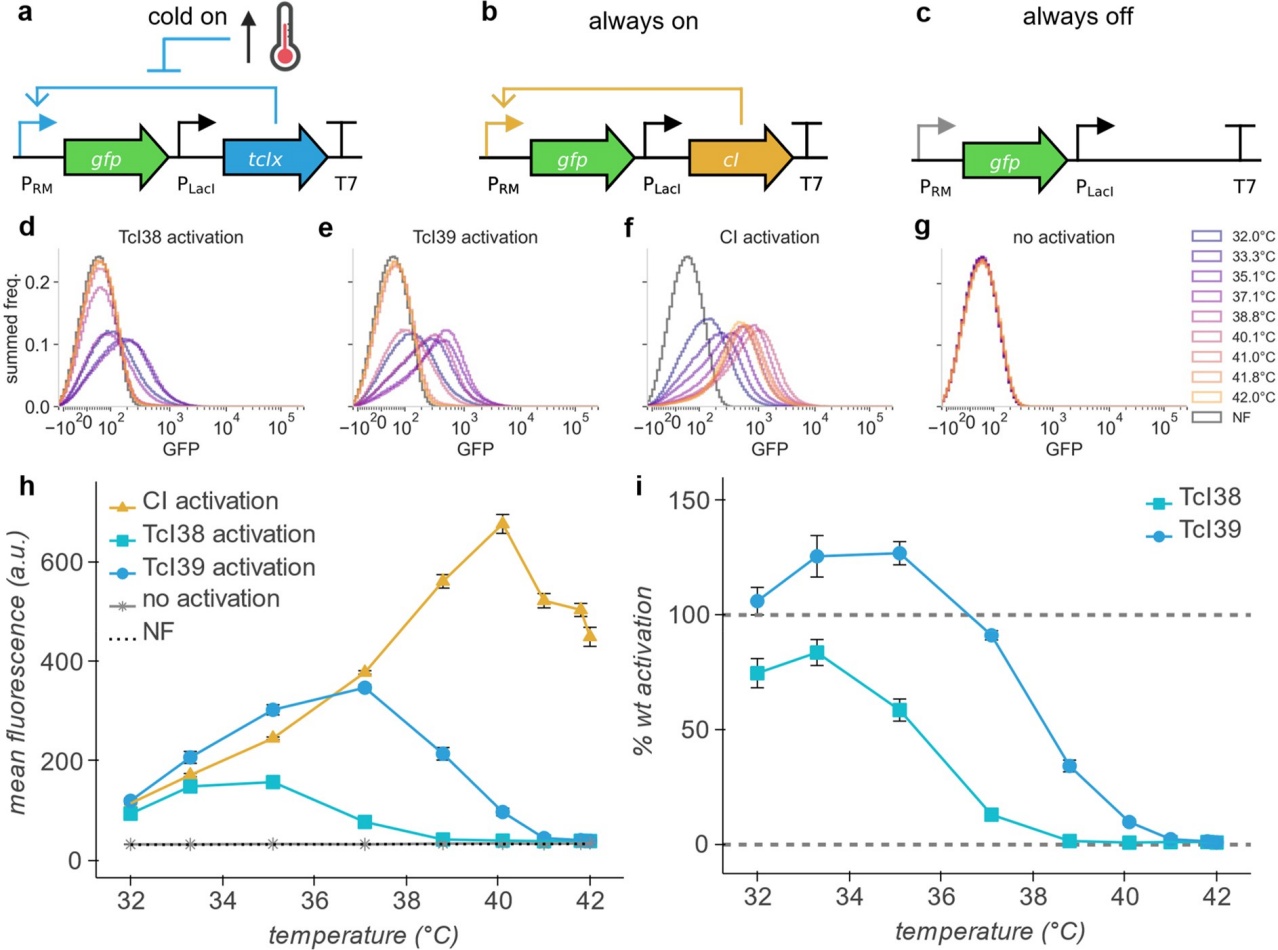
TcI mutants act as tunable, temperature-sensitive transactivators. (a,b,c) Circuit diagrams of gene activation constructs. TcI*x* (*x* = 38, 39) (a), wildtype CI (b), or no activator (c) activates expression of mWasabi (GFP) from the PRM promoter. (d,e,f,g) Summed frequency histograms for GFP channel for expression of GFP from PRM promoter by TcI38 (d), TcI39 (e), wildtype cI (f), or at baseline (no activator) (g). NF indicates nonfluorescent control measured in the same channel. (h) Thermal profile of mean population fluorescence of GFP expressed from the PRM promoter with activation by TcI38, TcI39, and wildtype CI, or at baseline (no activator) in *E. coli*. (i) Thermal profile of % wildtype activation of gene expression by TcI38 and TcI39. At each temperature, 100% wildtype activation indicates expression equal to wildtype CI, and 0% activation indicates expression equal to unactivated PRM. Eight hours of incubation, *n* = 4 biological replicates. Error bars represent ± SEM.

We quantified the gene expression level controlled by TcI38 or TcI39 via GFP fluorescence measured by flow cytometry in comparison with the level driven by wildtype CI or without an activator (Figure 1d,e,f,g). At 32 °C, each TcI mutant drives expression of GFP at levels greater than 75% of that generated by wildtype CI at the same temperature, and expression declines sharply with increasing temperature beyond a set threshold to a baseline equivalent to the nonfluorescent control (Figure 1h,i). TcI38 declines to 50% activation at 35.5 °C, while TcI39 reaches 50% activation at 38.5 °C. These effective transition temperatures are slightly downshifted from the midpoints observed when these proteins are acting as transcriptional repressors,^2^ suggesting that the interaction of TcI with its operator helps set its thermal set point.

After establishing the basic capabilities of tunable TcI activators, we endeavored to combine them with TcI repression. We assembled a construct wherein expression of GFP from the PRM promoter is activated by TcI39 and expression of mRFP1 (RFP) from the PR–PL tandem promoter is repressed by TcI39 (Figure 2a,b). We assayed the thermal response of this genetic circuit in *E. coli* via GFP and RFP fluorescence measured by flow cytometry (Figure 2c,d). Bivariate fluorescence analysis reveals that at intermediate temperatures, individual cells express both GFP and RFP. Mean hot-on RFP expression shows a sharp increase with temperature above 37 °C, consistent with previous work.^2^ Meanwhile, the mean cold-on GFP expression response is similar to the standalone TcI39 activation operon (Figure 1h), with slight upshifting of the transition temperature. We illustrated the differential expression of RFP and GFP above and below 39 °C using *E. coli* incubated at 37 and 44 °C (Figure 2e,f,g,h).

**Figure 2:**
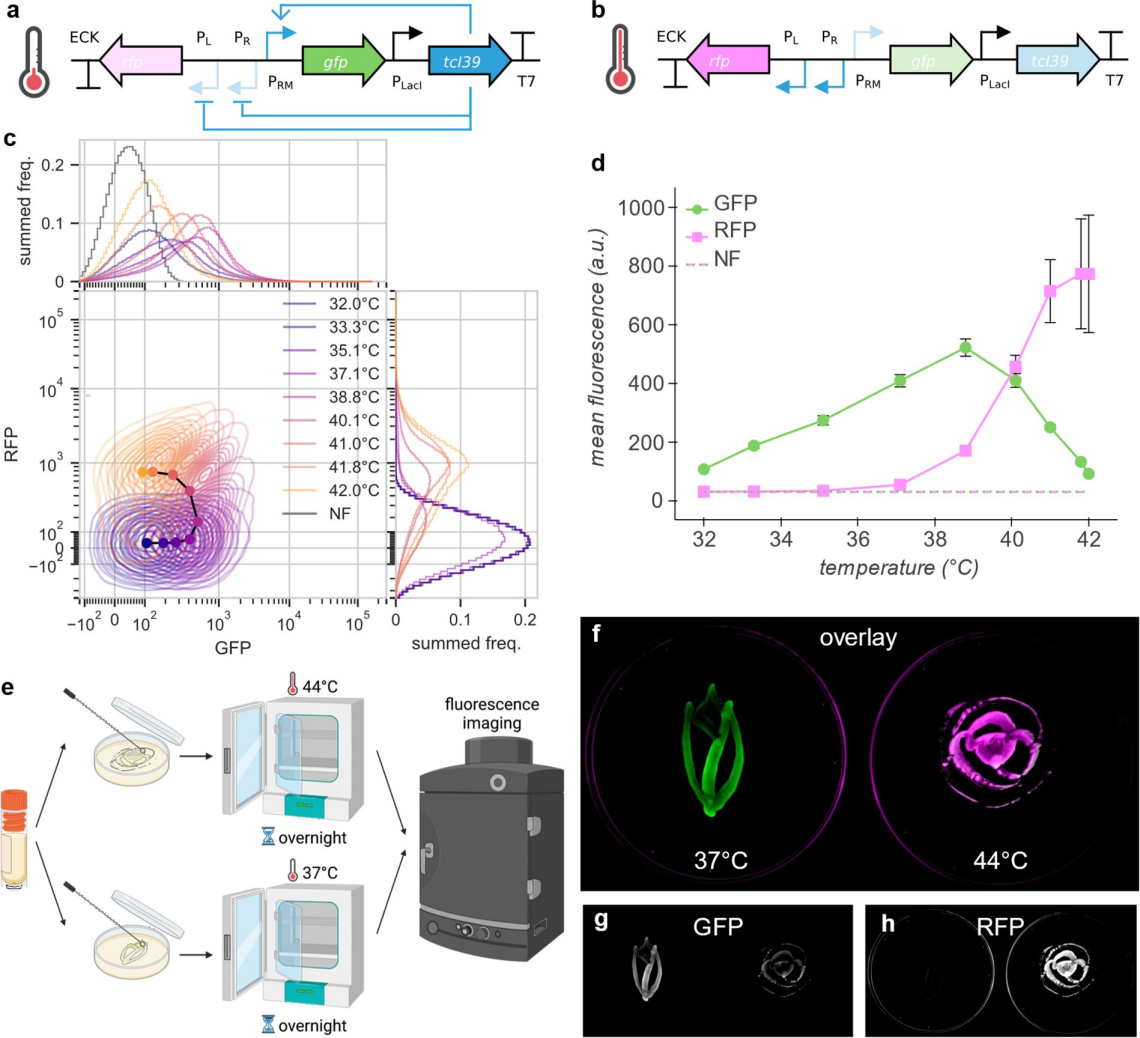
TcI39 simultaneously activates and represses, serving as a temperature-controlled state switch. (a,b) Circuit diagram of TcI39 switch construct with state of regulation arcs indicated at low (a) and high (b) temperature. TcI39 activates expression of mWasabi (GFP) from the PRM promoter and represses expression of mRFP1 (RFP) from the PR–PL tandem promoter. (c) Thermal profile of GFP and RFP co-expression. Central plot: bivariate kernel density estimation for RFP channel and GFP channel. Marginal plots: summed frequency histograms for RFP channel (right) and GFP channel (top). NF indicates nonfluorescent control measured in each channel (not shown in central plot for visual clarity; overlaps with 32.0, 33.3, 35.1 °C histograms in RFP channel). (d) Thermal profile of mean population fluorescence of GFP and RFP expressed in *E. coli* containing the TcI39 switch construct. Eight hours of incubation, *n* = 4 biological replicates. Error bars represent ± SEM. (e) Diagram of experiment illustrating differential gene expression with temperature. We drew images on two agar plates using a glycerol stock of *E. coli* containing the TcI state switch construct. We incubated each plate at a different temperature overnight before performing fluorescence imaging. (f) Overlay of GFP (green) and RFP (magenta) fluorescence images of *E. coli* containing the TcI switch construct, cultured on agar plates at 37 °C (left) and 44 °C (right). (g) GFP fluorescence image of plates in (f). (h) RFP fluorescence image of plates in (f). Color map limits were adjusted for each fluorophore to make the relative fluorescence levels of the two plates apparent. Parts of the figure were created with BioRender.com.

Our results establish the use of temperature-sensitive CI mutants as heat-inactivated transcriptional activators with tunable set points. In addition, due to the dual nature of these transcription factors, they can be used to control the expression of two genes in one circuit complementarily, with one expressed below the thermal set point, and the other above. The two TcI variants tested in this study operate at distinct set points within a range convenient for bacterial synthetic biology applications. The range of available set points could be further widened through directed evolution of TcI.^2^ Transcriptional activation by TcI mutants represents a *cool* new tool for global and local thermal control of cells.

## Supporting Information Available

Methods; Supplementary Tables S1–S2; Supplementary Figures S1–S13; and Supplementary References; including (i) genetic constructs and part sequences, (ii) flow cytometry gating on FSC/SSC, (iii) flow cytometry fluorescence data by biological replicate (PDF)
