# Supplementary material for: Tunable Temperature-Sensitive Transcriptional Activation Based on Lambda Repressor
Source: ACS Synth Biol. 2022 Jun 16;11(7):2518–22. doi: 10.1021/acssynbio.2c00093 (PMC9295150; doi:10.1021/acssynbio.2c00093)
Supplement: Supplementary file 1 — sb2c00093_si_001.pdf [file sb2c00093_si_001.pdf]

Supporting information

Tunable temperature-sensitive transcriptional activation based on Lambda repressor

Lealia L. Xiong[1], Michael A. Garrett[2], Marjorie T. Buss[2], Julia A. Kornfield[2], Mikhail G. Shapiro[2,3,*]

[1] Division of Engineering and Applied Sciences
[2] Division of Chemistry and Chemical Engineering
[3] Howard Hughes Medical Institute
California Institute of Technology
Pasadena, CA USA 91125

* Correspondence should be addressed to MGS: mikhail@caltech.edu

Table of contents
Methods
Supplementary Tables S1-S2
Supplementary Figures S1-S13
Supplementary References

METHODS

Plasmid construction and molecular biology
All plasmids were designed using SnapGene (GSL Biotech) and assembled via reagents from New England Biolabs for KLD mutagenesis (E0554S) or HiFi Assembly (E2621L). After assembly, constructs were transformed into NEB Turbo (C2984I) *E. coli* for growth and plasmid preparation. Thermal gene expression regulation assays were performed in NEB Stable *E. coli* (C3040I). Integrated DNA Technologies synthesized all PCR primers. TcI38, TcI39, and mWasabi[1] (GFP) were obtained from our previous work[2]. mWasabi was tagged at the C-terminus with the DAS ssrA tag[3] (amino acid sequence AANDENYADAS). mRFP1[4] (RFP) was obtained from the pTU1-A-RFP plasmid[5], a gift from Paul Freemont (Addgene plasmid # 72939 ; http://n2t.net/addgene:72939 ; RRID:Addgene_72939). The transcriptional terminator referred to in Figure 2 as ECK is the ECK120029600 terminator[6] and was synthesized as a gBlock by Integrated DNA Technologies. Gene circuit diagrams were created using the DNAplotlib[7] library in Python.

Thermal regulation assays
Determination of temperature-dependent gene expression was performed using slight modifications from a previously-described method[2]. 1 mL cultures of 2x YT medium with 100 μg/mL ampicillin were inoculated with a single colony per culture and grown at 30°C, 250 rpm for 20 h. After dilution to OD600 = 0.1 in 2 mL LB (Sigma) with 100 μg/mL ampicillin, the cells were propagated at 30°C, 250 rpm until reaching OD600 = 0.25 as measured using a Nanodrop 2000c (Thermo Scientific) in cuvette mode. The cultures were dispensed in 50 μL aliquots into 8-well PCR strips (Bio-Rad) and incubated for 8 h in a thermal gradient using a DNA Engine Tetrad 2 Peltier Thermal Cycler (Bio-Rad) with the lid set to 50°C. After thermal stimulus, cultures were immediately diluted 100-fold into PBS with 0.5% BSA and 1 mg/mL chloramphenicol and chilled on ice to stop protein expression.

Cell fluorescence was measured using a MACSQuant VYB flow cytometer (Miltenyi Biotec) with appropriate settings: FSC 400 V, SSC 250 V, Y2 (dsRed/txRed) 550 V, B1 (GFP/FITC) 520 V. At least 40,000 events were collected for each sample. NEB Stable *E. coli* transformed with the pTlpA-wasabi-NF

plasmid (obtained from our previous work[2]) served as a non-fluorescent control. For each figure, all biological replicates and samples were measured in the same flow cytometry session.

All data analysis was performed using custom Python scripts with some functions from the Cytoflow[8] package. The geometric mean of fluorescence intensity was calculated using a bi-geometrical approach for negative and positive values[9]. Percent of wildtype activation was determined according to equation (1):

$$\% \text{ activation} = \frac{\text{avg } F - \text{avg } F_{\text{no CI}}}{\text{avg } F_{\text{CI}} - \text{avg } F_{\text{no CI}}} \tag{1}$$

Here, F is the geometric mean of fluorescence of a given sample at a given temperature, and avg F refers to the mean of 4 biological replicates. 1D and 2D histograms from biological replicates were combined for each circuit and temperature after weighting each count according to the total number of counts in its replicate.1D histogram bins were chosen as the minimum of the number of bins given by the Freedman-Diaconis rule for each replicate for each circuit and temperature, with a lower limit of 100 bins. 2D histograms used 50 bins in each dimension. Bivariate kernel density estimation was performed using Gauusian kernels with bandwidths selected by Scott's rule, clipping evaluation to values below 1e4 for the GFP channel and values below 8e3 for the RFP channel. Logicle scale[10,11] parameters were calculated using Cytoflow[12]. All data were plotted in Python using the Holoviews, Bokeh, and Matplotlib packages.

Plated illustrations of gene expression

Images were drawn on LB agar (Sigma) plates with 100 μg/mL ampicillin using a glycerol stock of *E. coli* containing the genetic circuit of interest. After incubation overnight at the temperatures of interest, the plates were imaged using a ChemiDoc MP Gel Imaging System (Bio-Rad). Filters used for RFP and GFP were the 530/28 filter and 605/50 filter, respectively. Colormap adjustments and color channel overlays were performed using ImageJ software[13].

Data and code availability

Plasmids will be made available through Addgene upon publication. Data analysis code will be made available on Github (https://github.com/shapiro-lab) upon publication. All other materials and data are available from the corresponding author upon reasonable request.

SUPPLEMENTARY TABLES S1-S2

Supplementary Table S1. Genetic constructs used in this study.

Plasmid	Transcriptional Regulator	Output Gene Product(s)
pCIwt-PRM-Wasabi	CI	mWasabi
pTcI38-PRM-wasabi	TcI38	mWasabi
pTcI39-PRM-wasabi	TcI39	mWasabi
pBaseline-PRM-wasabi	none	mWasabi
pTcI39-state-switch	TcI39	mWasabi, mRFP1
pTlpA-wasabi-NF	TlpA	Nonfluorescent mWasabi (S71T, G73A)

Supplementary Table S2. Sequences of DNA parts used in this study.

Type	Name	Sequence
promoter	PRM/PR mutOR3	ATTATCACCGCCAGAGGTAAAATAGTCAACACGCACGGTGTTAGATAT<br>TTATAAATAGTGGTGATAGATT
promoter	PL	AACCATCTGCGGTGATAAATTATCTCTGGCGGTGTTGACATAAATACCACTG<br>GCGGTGATACTGAGCACATCAGCAGG
promoter	PLacI	GACACCATCGAATGGCGCAAACCTTTCGCGGTATGGCATGATAGCGCCCGGA<br>AGAGAGTCAATTCAGGGTGGTGAAT
degradation tag	DAS ssrA tag	GCTGCTAACGACGAAAACTACGCTGACGCTTCT
terminator	T7	CTAGCATAACCCCTTGGGGCCTCTAAACGGGTCTTGAGGGGTTTTTTG
terminator	ECK120029600	TTCAGCCAAAAAACTTAAGACCGCCGGTCTTGTCCACTACCTTGCAGTAATG<br>CGGTGGACAGGATCGGCGGTTTTCTTTTCTCTTCTCAA
gene	CI	ATGAGCACAAAAAAGAAACCATTAACACAAGAGCAGCTTGAGGACGCACGTC<br>GCCTTAAAGCAATTTATGAAAAAAAGAAAAATGAACTTGGCTTATCCCAGGAA<br>TCTGTCGCAGACAAGATGGGGATGGGGCAGTCAGGCGTTGGTGCTTTATTT<br>AATGGCATCAATGCATTAAATGCTTATAACGCCGCATTGCTTGCAAAAATTCT<br>CAAAGTTAGCGTTGAAGAATTTAGCCCTTCAATCGCCAGAGAAATCTACGAG<br>ATGTATGAAGCGGTTAGTATGCAGCCGTCACTTAGAAGTGAGTATGAGTACC<br>CTGTTTTTTCTCATGTTCAGGCAGGGATGTTCTCACCTGAGCTTAGAACCTT<br>TACCAAAGGTGATGCGGAGAGATGGGTAAGCACAACCAAAAAAGCCAGTGAT<br>TCTGCATTCTGGCTTGAGGTTGAAGGTAATTCCATGACCGCACCAACAGGCT<br>CCAAGCCAAGCTTTCCTGACGGAATGTTAATTCTCGTTGACCCTGAGCAGGC<br>TGTTGAGCCAGGTGATTTCTGCATAGCCAGACTTGGGGGTGATGAGTTTAC<br>CTTCAAGAAACTGATCAGGGATAGCGGTCAGGTGTTTTTACAACCACTAAAC<br>CCACAGTACCCAATGATCCCATGCAATGAGAGTTGTTCCGTTGTGGGGAAAG<br>TTATCGCTAGTCAGTGGCCTGAAGAGACGTTTGGCTGA
gene	TcI38	ATGAGCACAAAAAAGAAACCATTAACACAAGAGCAGCTTGAGGACGCACGTC<br>GCCTTAAAGCAATTTATGAAAAAAAGAAAAATGAACTTGGCTTATCCCAGGAA<br>TCTGTCGCAGACAAGATGGGGATGGGGCAGTCAGGCGTTGGTGCCTTATTT<br>AATGGCATCAATGCATTAAATGCTTATAACGCCGCATCGCTTACAAGAATTCT<br>CAAAGTTAGCGTTGAAGAATTTAGCCCTTCAATCGCCAGAGAAATCTACGAG<br>ATGTATGAAGCGGTTAGTATGCAGCCGTCACTTAGAAGTGAGTATGAGTACC<br>CTGTTTTTTCTCATGTTCAGGCAGGGATGCTCTCACCTGAGCTTAGAACCTT<br>TACCAAAGGTGGTGCGGAAAGGTGGGTAAGCACAACCAAAAAAGCCAGTGAT<br>TCTGCATTCTGGCTTGAGGTTGAAGGTAATTCCATGACAGCACCAACAGGCT<br>CCAAGCCAAGCTTTCCTGACGGAATGTTAATTCTCGTTGACCCTGAGCAGGC<br>TGTTGAGCCAGGTGATTTCTGCATAGCCAGACTCGGGGGTGGTGAGTTTAC<br>CTTCAAGAAACTGATCAGGGATAGCGGTCAGGTGTTTTTACAACCACTAAAC<br>CCACAGTACCCAATGATCCCATGCAATGAGAGTTGTTCCGTTGTGGGGAAAG<br>TTATCGCTAGTCAGTGGCCTGAAGAGACGTTTGGCTGA
gene	TcI39	ATGAGCACAAAAAAGAAACCATTAACACAAGAGCAGCTTGAGGACGCACGTC<br>GCCTTAAAGCAATTTATGAAAAAAAGAAAAATGAACTTGGCTTATCCCAGGAA<br>TCTGTCGCAGACAAGATGGGGATGGGGCAGTCAGGCGTTGGTGCTTTATTT<br>AATGGCATCAATGCATTAAATGCTTATAACGCCGCATTGCTTACAAAAATTCT<br>CAAAGTTAGCGTTGAAGAATTTAGCCCTTCAATCGCCAGAGAAATCTACGAG<br>ATGTATGAAGCGGTTAGTATGCAGCCGTCACTTAGAAGTGAGTATGAGTACC<br>CTGTTTTTTCTCATGTTCAGGCAGGGATGTTCTCACCTGAGCTTAGAACCTT<br>TACCAAAGGTGATGCGGAGAGATGGGTAAGCACAACCAAAAAAGCCAGTGAT<br>TCTGCATTCTGGCTTGAGGTTGAAGGTAATTCCATGACCGCACCAACAGGCT<br>CCAAGCCAAGCTTTCCTGACGGAATGTTAATTCTCGTTGACCCTGAGCAGGC<br>TGTTGAGCCAGGTGATTTCTGCATAGCCAGACTTGGGGGTGATGAGTTTAC<br>CTTCAAGAAACTGATCAGGGATAGCGGTCAGGTGTTTTTACAACCACTAAAC<br>CCACAGTACCCAATGATCCCATGCAATGAGAGTTGTTCCGTTGTGGGGAAAG<br>TTATCGCTAGTCAGTGGCCTGAAGAGACGTTTGGCTGA

gene	TlpA	ATGCGTCCGGCGACATACGAACCAGAACAGATTATTGAAGCAGGGCTGGCCC<br>TGCAGGCTGAAGGACGGAATATCACCGGGTTCGCACTACGTAACCAGGTGG<br>GTGGCGGCAATCCGACACGTCTCCGCCAGATATGGGACGAATACCAGGCTT<br>CACAGAGCACGGTCGTCACTGAACCCGTTGCCGAGCTGCCAGTGGAAGTGG<br>CTGAAGAAGTGAAGGCCGTCTCCGCCGCGCTGTCCGAACGCATCACCCAGC<br>TGGCGACAGAACTGAATGACAAGGCGGTCCGGGCTGCAGAACGCCGGGTTG<br>CGGAAGTCACGCGTGCTGCCGGTGAACAGACCGCACAGGCAGAGCGGGAGC<br>TGGCCGACGCCGCGCAGACAGTCGACGACCTGGAAGAAAAACTGGATGAACT<br>GCAGGACAGATATGACAGTTTGACGCTGGCGCTGGAGTCAGAACGTTCACT<br>GCGTCAGCAGCATGATGTGGAGATGGCCCAGCTGAAAGAGCGTCTTGCGGC<br>CGCTGAAGAGAATACCCGTCAGCGAGAGGAACGGTATCAGGAGCAGAAGACA<br>GTGCTGCAGGATGCGCTTAATGCGGAGCAGGCACAGCACAAAAACACGCGG<br>GAAGACCTGCAGAAACGACTGGAGCAAATTTCTGCCGAAGCTAATGCGCGTA<br>CAGAAGAACTGAAGTCTGAACGCGATAAAGTCAATACTCTCCTTACCCGCCT<br>TGAATCGCAGGAAAATGCGCTGGCCTCAGAACGTCAGCAGCATCTGGCCACC<br>CGCGAAACGCTGCAGCAACGCCTCGAGCAGGCCATCGCTGACACGCAGGCG<br>CGCGCCGGTGAGATTGCACTTGAACGTGACAGAGTCAGCAGCCTCACCGCAA<br>GGCTGGAATCGCAGGAAAAGGCCTCCTCGGAGCAACTGGTGCGTATGGGCA<br>GTGAAATAGCCAGTCTGACAGAGCGTTGCACACAGCTGGAAAACCAGCGTGA<br>TGATGCCCGTCTGGAGACGATGGGGGAGAAAGAAACGGTCGCGGCACTGCG<br>TGGTGAGGCTGAAGCCCTGAAGCGTCAGAACCAGTCACTGATGGCGGCGCT<br>TTCAGGCAATAAACAGACCGGTGGCCAGAATGCGTTAA
gene	mWasabi	ATGGTGAGCAAGGGCGAGGAGACCACAATGGGCGTAATCAAGCCCGACATG<br>AAGATCAAGCTGAAGATGGAGGGCAACGTGAATGGCCACGCCTTCGTGATCG<br>AGGGCGAGGGCGAGGGCAAGCCCTACGACGGCACCAACACCATCAACCTGG<br>AGGTGAAGGAGGGAGCCCCCCTGCCCTTCTCCTACGACATTCTGACCACCGC<br>GTTCAGTTACGGCAACAGGGCCTTCACCAAGTACCCCGACGACATCCCCAAC<br>TACTTCAAGCAGTCCTTCCCCGAGGGCTACTCTTGGGAGCGCACCATGACCT<br>TCGAGGACAAGGGCATCGTGAAGGTGAAGTCCGACATCTCCATGGAGGAGG<br>ACTCCTTCATCTACGAGATACACCTCAAGGGCGAGAACTTCCCCCCCAACGG<br>CCCCGTGATGCAGAAGGAGACCACCGGCTGGGACGCCTCCACCGAGAGGAT<br>GTACGTGCGCGACGGCGTGCTGAAGGGCGACGTCAAGATGAAGCTGCTGCT<br>GGAGGGCGGCGGCCACCACCGCGTTGACTTCAAGACCATCTACAGGGCCAA<br>GAAGGCGGTGAAGCTGCCCGACTATCACTTTGTGGACCACCGCATCGAGATC<br>CTGAACCACGACAAGGACTACAACAAGGTGACCGTTTACGAGATCGCCGTGG<br>CCCGCAACTCCACCGACGGCATGGACGAGCTGTACAAGGGCTGA
gene	mRFP1	ATGGCTTCCTCCGAAGACGTTATCAAAGAGTTCATGCGTTTCAAAGTTCGTA<br>TGGAAGGTTCCGTTAACGGTCACGAGTTCGAAATCGAAGGTGAAGGTGAAG<br>GTCGTCCGTACGAAGGTACCCAGACCGCTAAACTGAAAGTTACCAAAGGTGG<br>TCCGCTGCCGTTCGCTTGGGACATCCTGTCCCCGCAGTTCCAGTACGGTTC<br>CAAAGCTTACGTTAAACACCCGGCTGACATCCCGGACTACCTGAAACTGTCC<br>TTCCCGGAAGGTTTCAAATGGGAACGTGTTATGAACTTCGAAGACGGTGGTG<br>TTGTTACCGTTACCCAGGACTCCTCCCTGCAAGACGGTGAGTTCATCTACAA<br>AGTTAAACTGCGTGGTACCAACTTCCCGTCCGACGGTCCGGTTATGCAGAAA<br>AAAACCATGGGTTGGGAAGCTTCCACCGAACGTATGTACCCGGAAGACGGTG<br>CTCTGAAAGGTGAAATCAAAATGCGTCTGAAACTGAAAGACGGTGGTCACTA<br>CGACGCTGAAGTTAAAACCACCTACATGGCTAAAAAACCGGTTCAGCTGCCG<br>GGTGCTTACAAAACCGACATCAAACTGGACATCACCTCCCACAACGAAGACT<br>ACACCATCGTTGAACAGTACGAACGTGCTGAAGGTCGTCACTCCACCGGTGC<br>TTAA
gene	mWasabi-NF	ATGGTGAGCAAGGGCGAGGAGACCACAATGGGCGTAATCAAGCCCGACATG<br>AAGATCAAGCTGAAGATGGAGGGCAACGTGAATGGCCACGCCTTCGTGATCG<br>AGGGCGAGGGCGAGGGCAAGCCCTACGACGGCACCAACACCATCAACCTGG<br>AGGTGAAGGAGGGAGCCCCCCTGCCCTTCTCCTACGACATTCTGACCACCGC<br>GTTCACCTACGCCAACAGGGCCTTCACCAAGTACCCCGACGACATCCCCAAC<br>TACTTCAAGCAGTCCTTCCCCGAGGGCTACTCTTGGGAGCGCACCATGACCT<br>TCGAGGACAAGGGCATCGTGAAGGTGAAGTCCGACATCTCCATGGAGGAGG<br>ACTCCTTCATCTACGAGATACACCTCAAGGGCGAGAACTTCCCCCCCAACGG<br>CCCCGTGATGCAGAAGGAGACCACCGGCTGGGACGCCTCCACCGAGAGGAT<br>GTACGTGCGCGACGGCGTGCTGAAGGGCGACGTCAAGATGAAGCTGCTGCT<br>GGAGGGCGGCGGCCACCACCGCGTTGACTTCAAGACCATCTACAGGGCCAA<br>GAAGGCGGTGAAGCTGCCCGACTATCACTTTGTGGACCACCGCATCGAGATC<br>CTGAACCACGACAAGGACTACAACAAGGTGACCGTTTACGAGATCGCCGTGG<br>CCCGCAACTCCACCGACGGCATGGACGAGCTGTACAAGGGCTGA

SUPPLEMENTARY FIGURES S1-S13

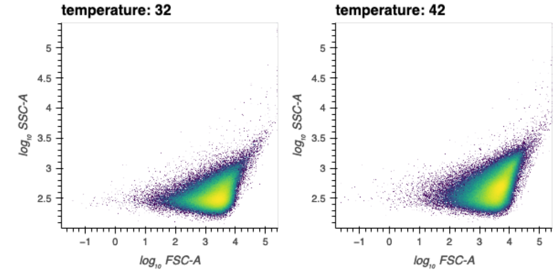

Supplementary figure S1. Forward scatter area (FSC-A) vs. side scatter area (SSC-A) for nonfluorescent control used to compare to gene expression from the PRM promoter at baseline (no activator) and with activation by TcI38, TcI39, wildtype CI (pooled data from 4 biological replicates).

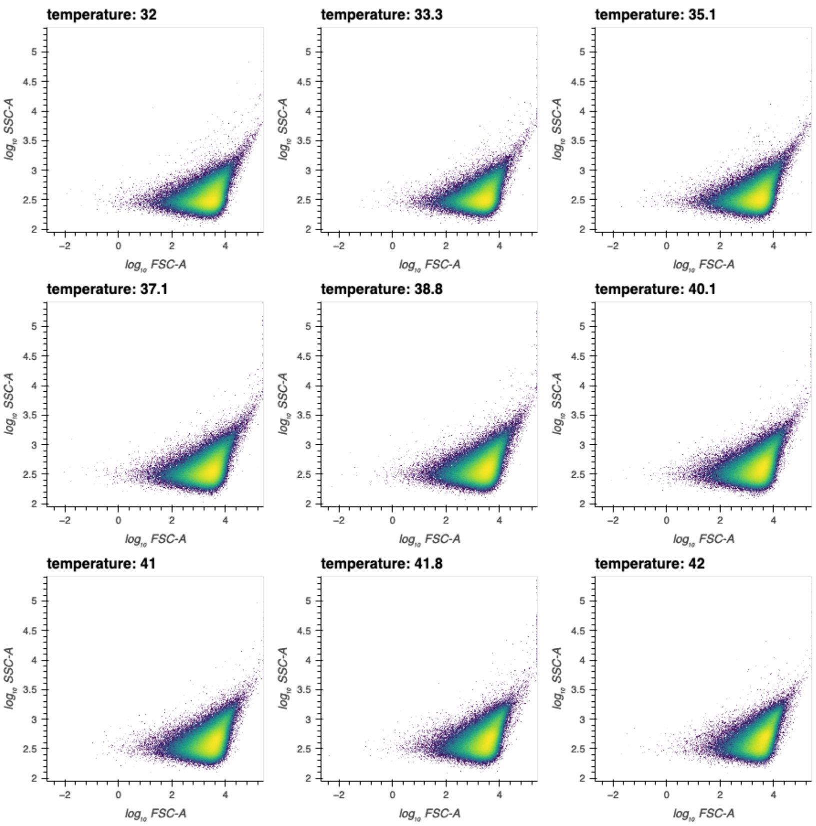

Supplementary figure S2. Forward scatter area (FSC-A) vs. side scatter area (SSC-A) for TcI38 activation samples (pooled data from 4 biological replicates).

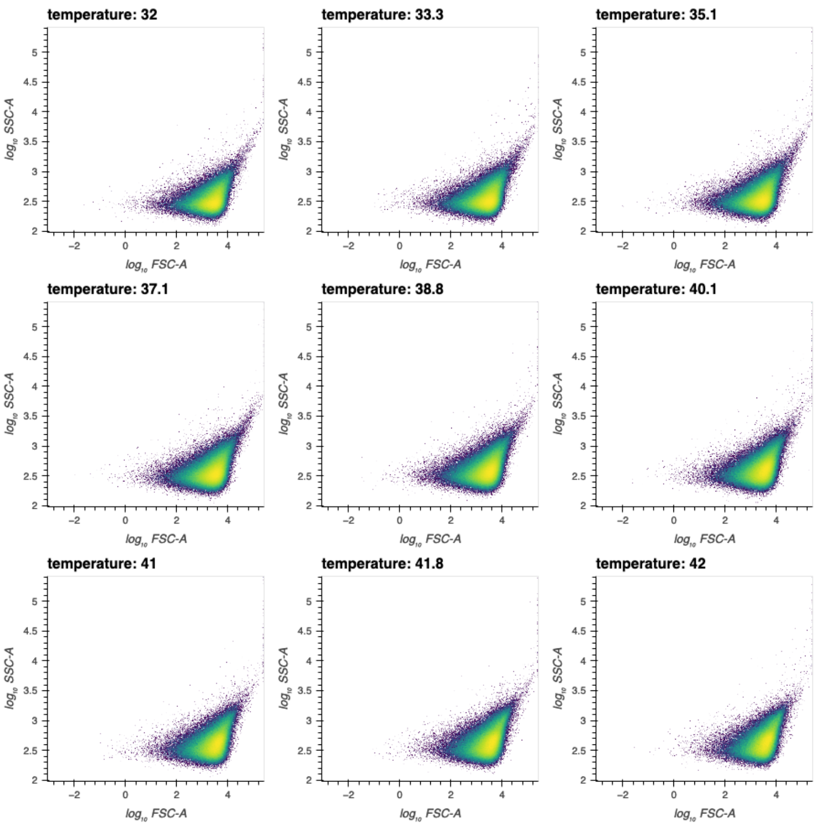

Supplementary figure S3. Forward scatter area (FSC-A) vs. side scatter area (SSC-A) for TcI39 activation samples (pooled data from 4 biological replicates).

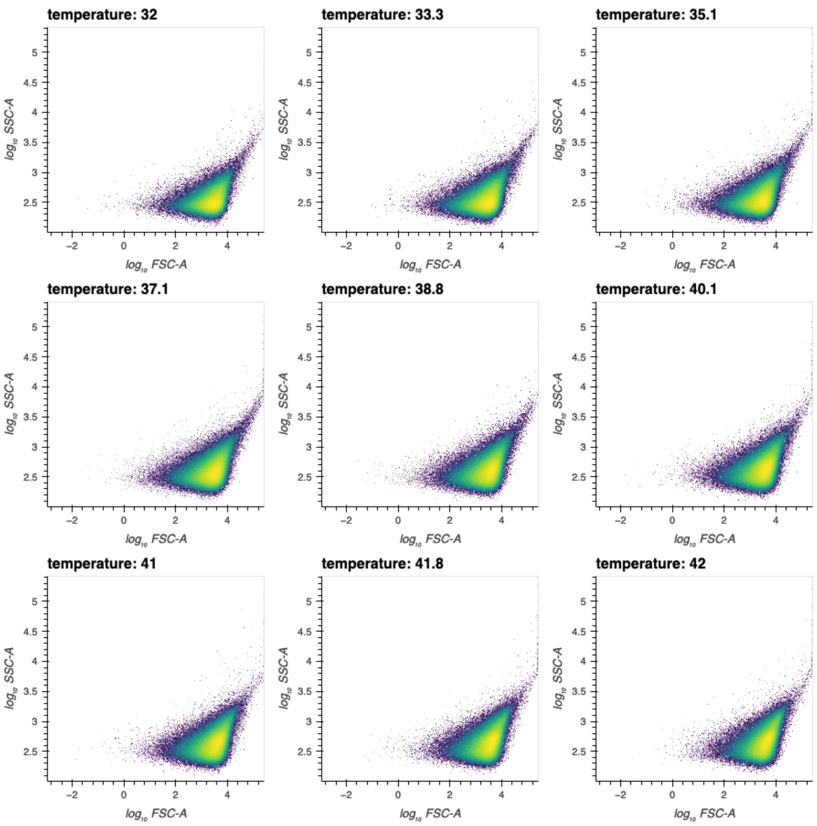

Supplementary figure S4. Forward scatter area (FSC-A) vs. side scatter area (SSC-A) for no activation samples (pooled data from 4 biological replicates).

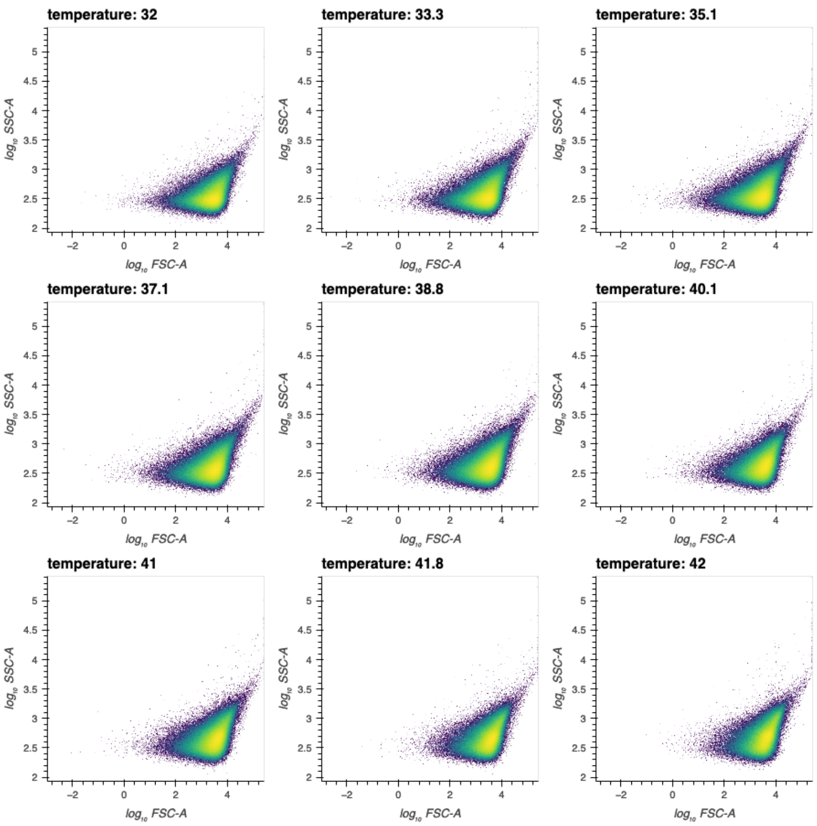

Supplementary figure S5. Forward scatter area (FSC-A) vs. side scatter area (SSC-A) for wildtype CI activation samples (pooled data from 4 biological replicates.

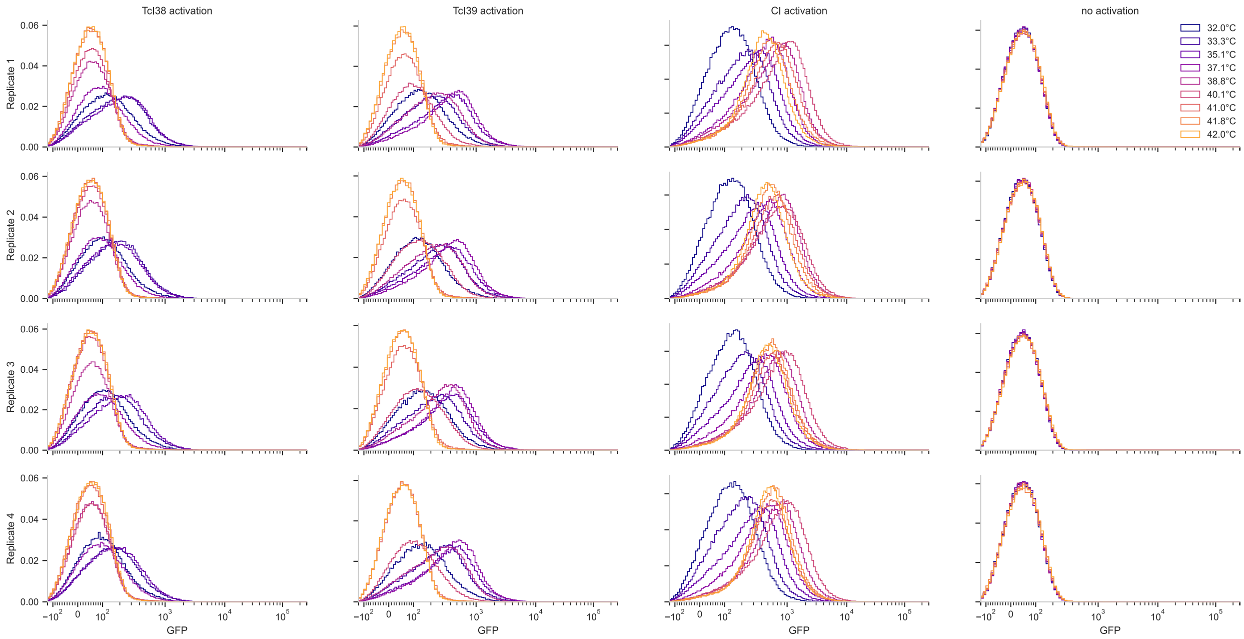

Supplementary figure S6. Frequency histograms for GFP channel for expression of mWasabi from PRM promoter by TcI38 (column 1), TcI39 (column 2), wildtype cI (column 3), or at baseline (no activator) (column 4), for each biological replicate (rows). Number of bins for each histogram determined by Freedman-Diaconis rule, with a minimum of 100 bins.

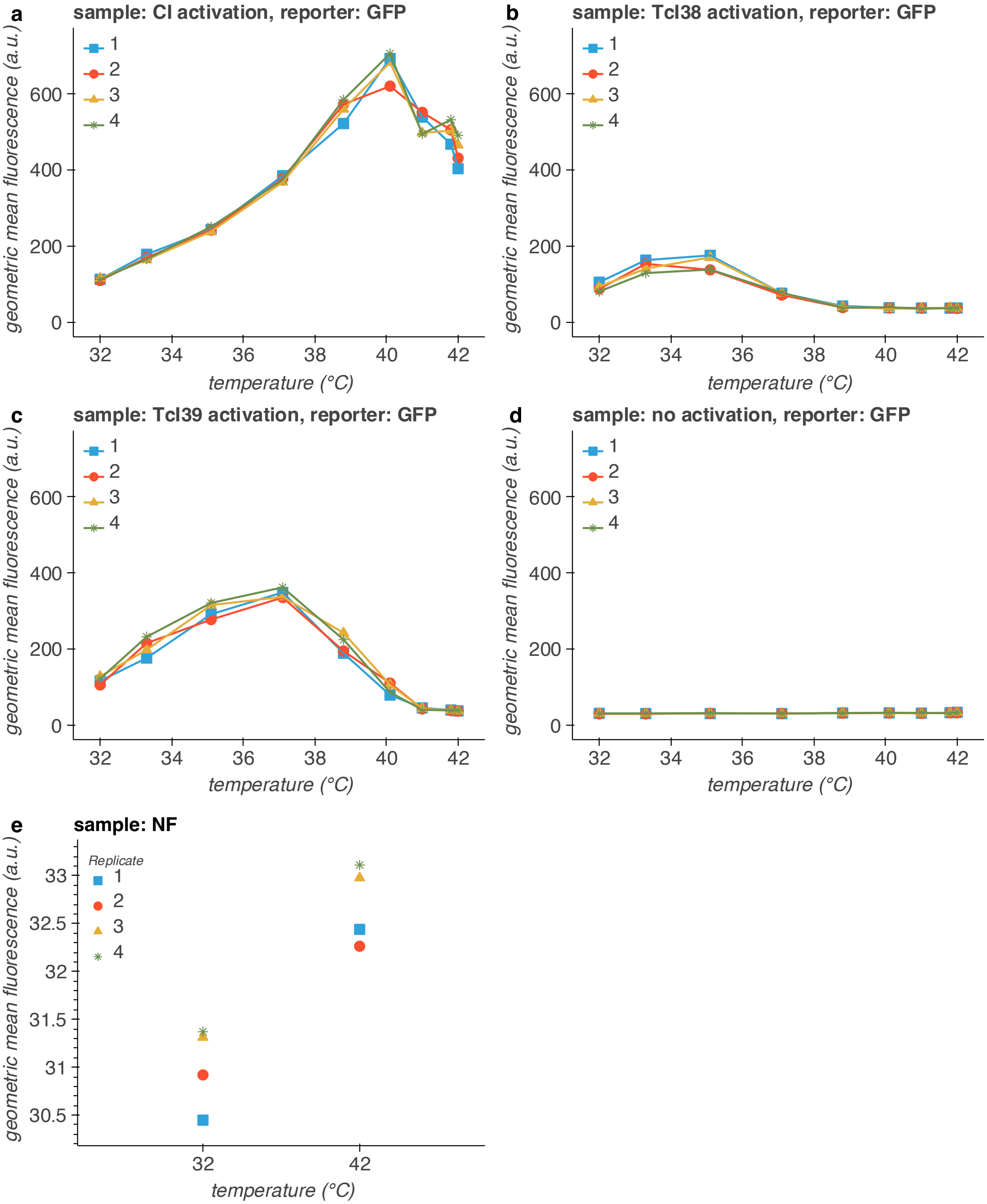

Supplementary figure S7. (a-c) Geometric mean of fluorescence in GFP channel for expression of mWasabi from PRM promoter by TcI38 **(a)**, TcI39 **(b)**, cI **(c)**, or at baseline (no activator) **(d)**, for each biological replicate. **(e)** Geometric mean of fluorescence in GFP channel for each biological replicate of nonfluorescent control.

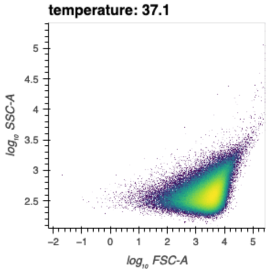

Supplementary figure S8. Forward scatter area (FSC-A) vs. side scatter area (SSC-A) for nonfluorescent control used to compare to gene expression by the TcI39 state switch construct (pooled data from 4 biological replicates).

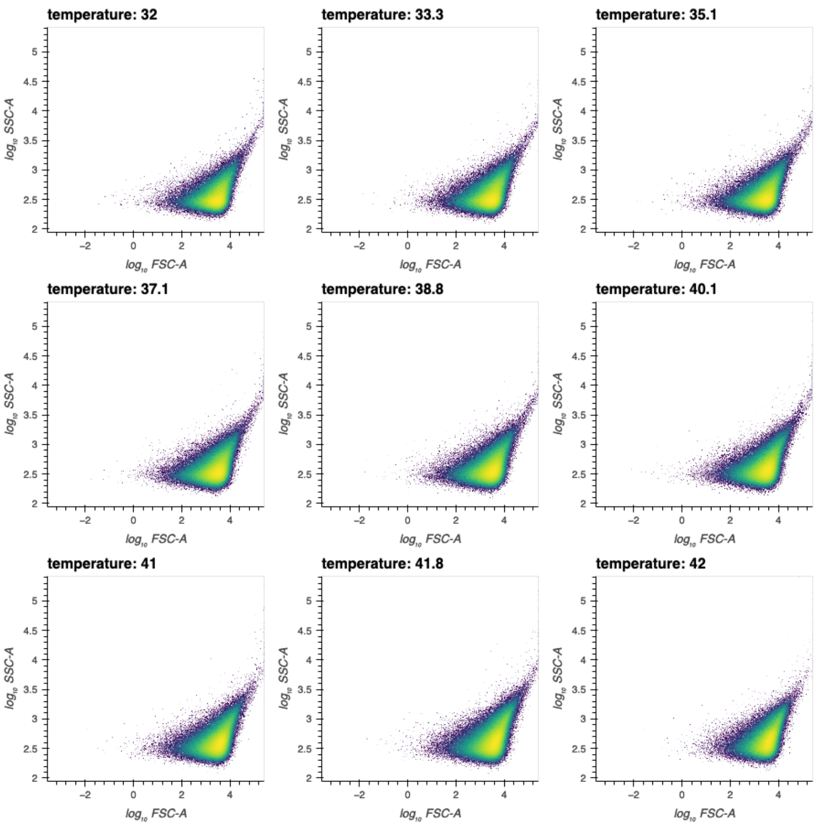

Supplementary figure S9. Forward scatter area (FSC-A) vs. side scatter area (SSC-A) for TcI39 state switch samples (pooled data from 4 biological replicates).

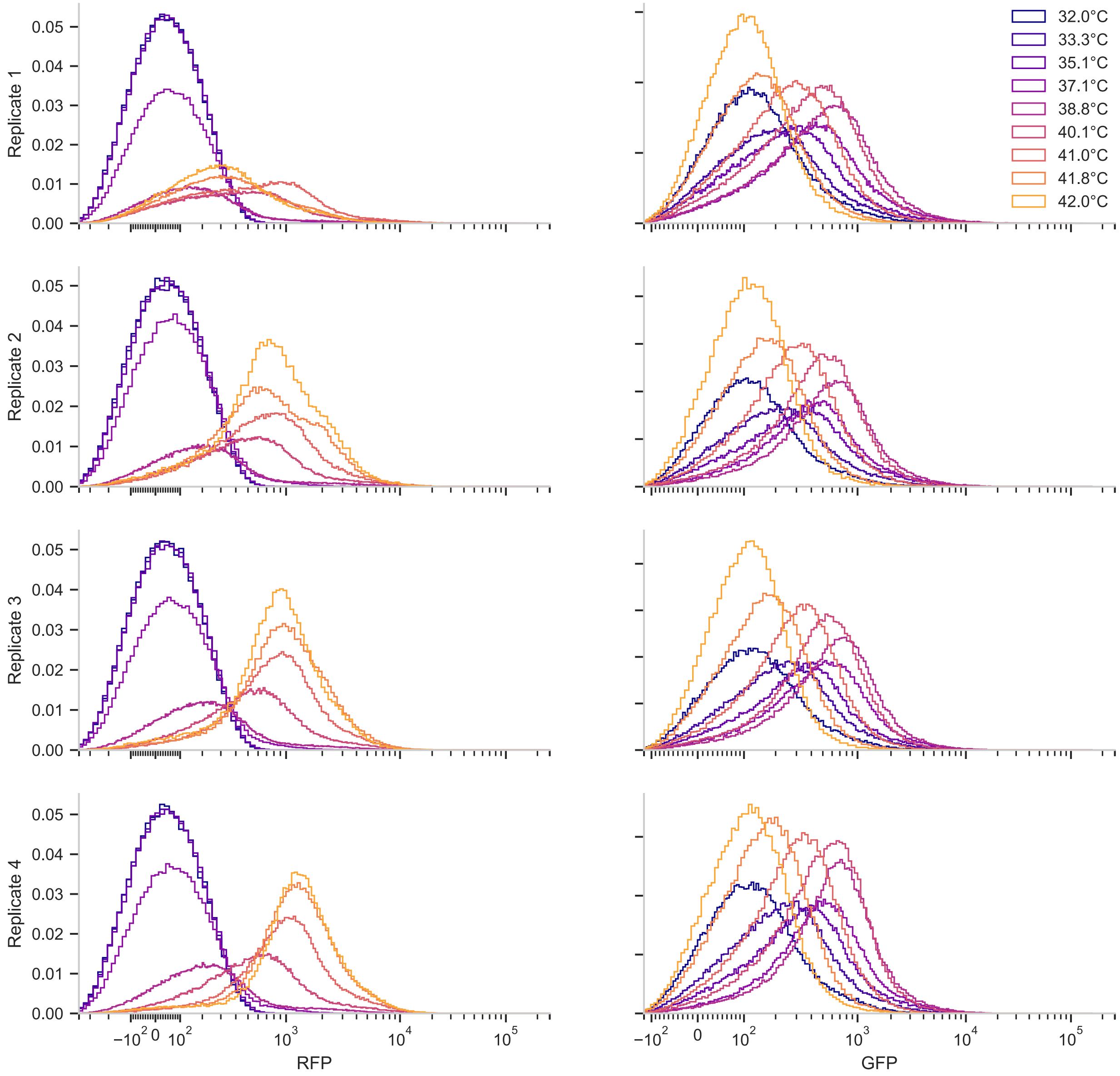

Supplementary figure S10. Frequency histograms for RFP channel (left) and GFP channel (right) for expression by TcI39 state switch construct, for each biological replicate (rows). Number of bins for each histogram determined by Freedman-Diaconis rule, with a minimum of 100 bins.

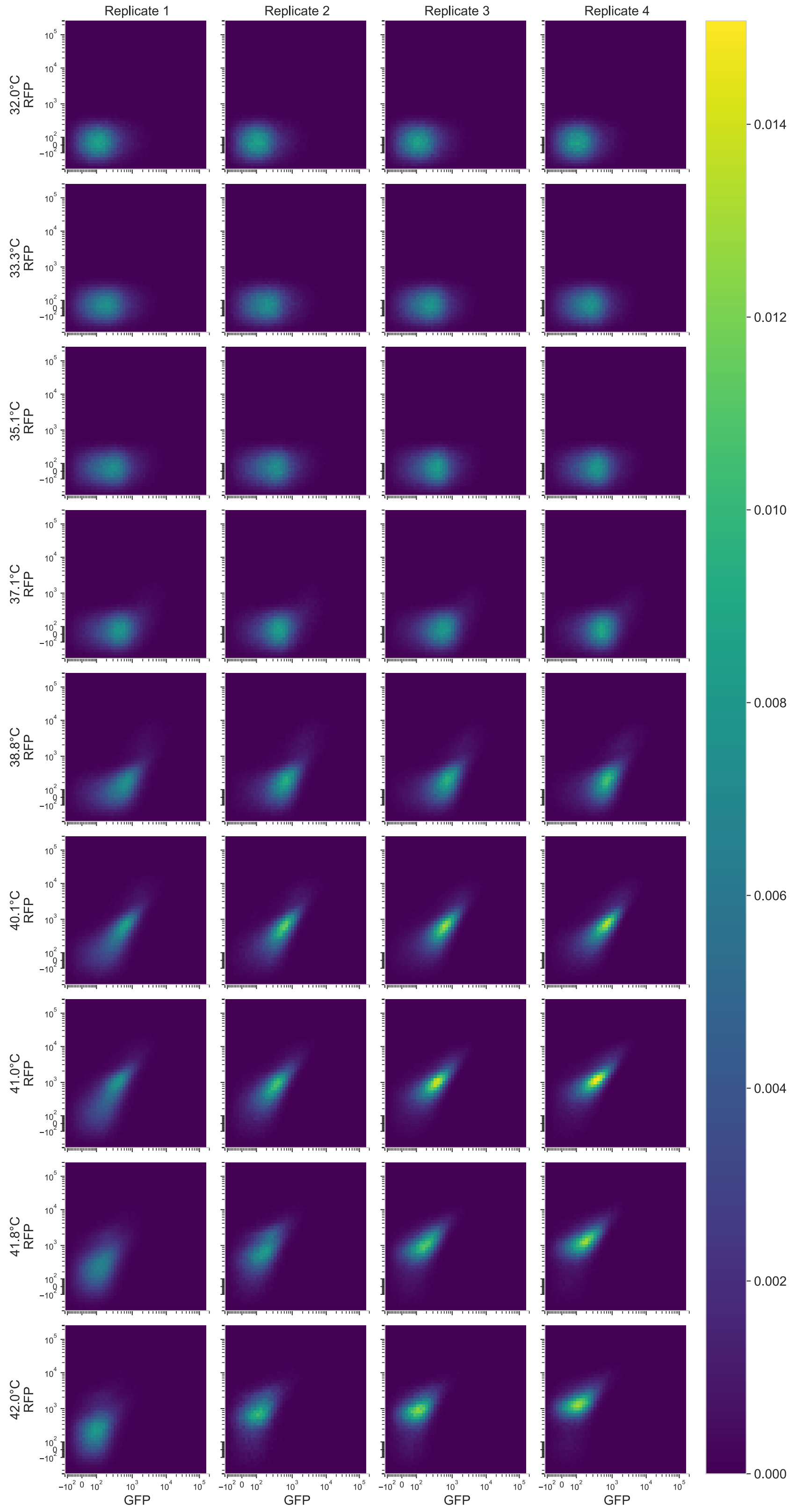

Supplementary figure S11. 2D frequency histograms for RFP channel and GFP channel for expression by TcI39 construct, for each biological replicate (columns). 50 bins in each dimension.

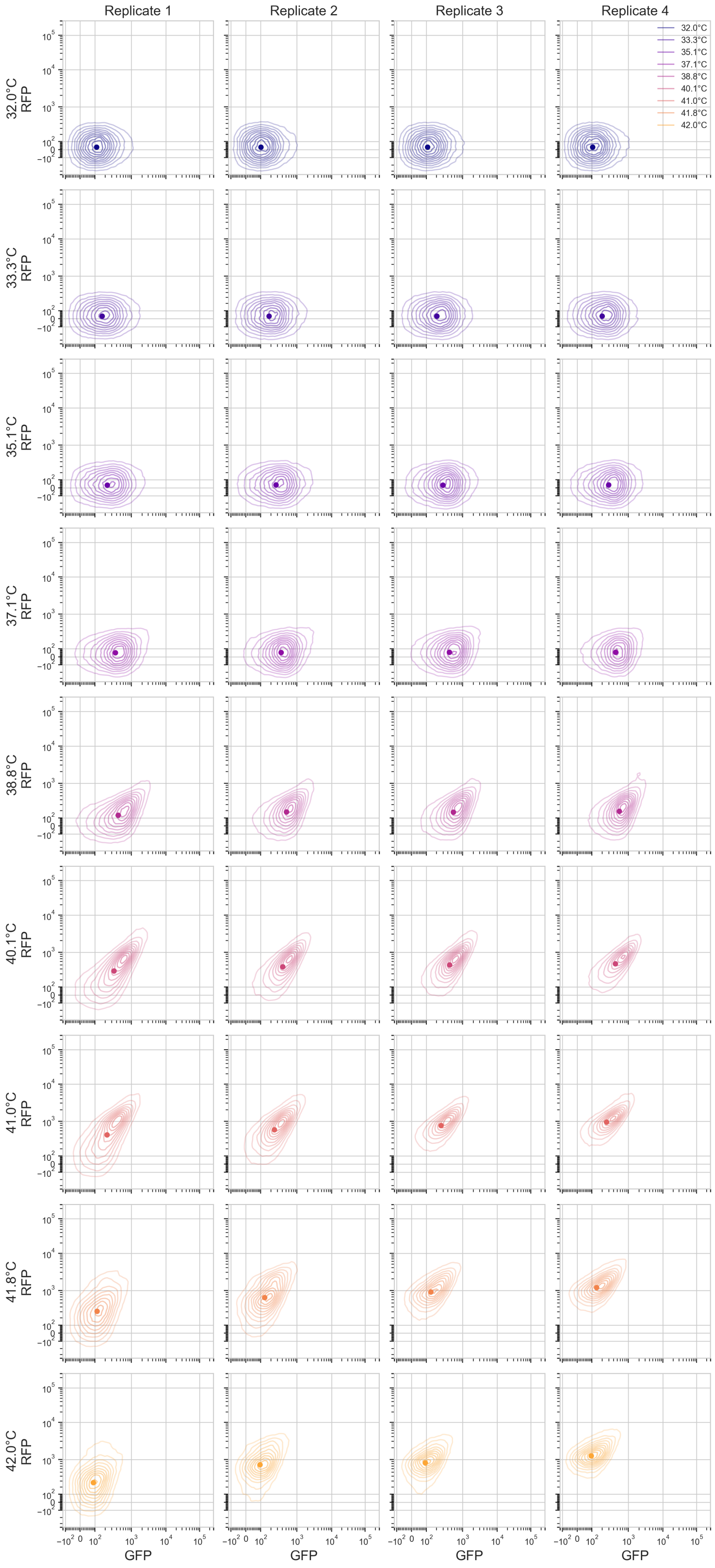

Supplementary figures S12. Bivariate kernel density estimation for RFP channel and GFP channel for expression by TcI39 construct, for each biological replicate (columns). 50 bins in each dimension.

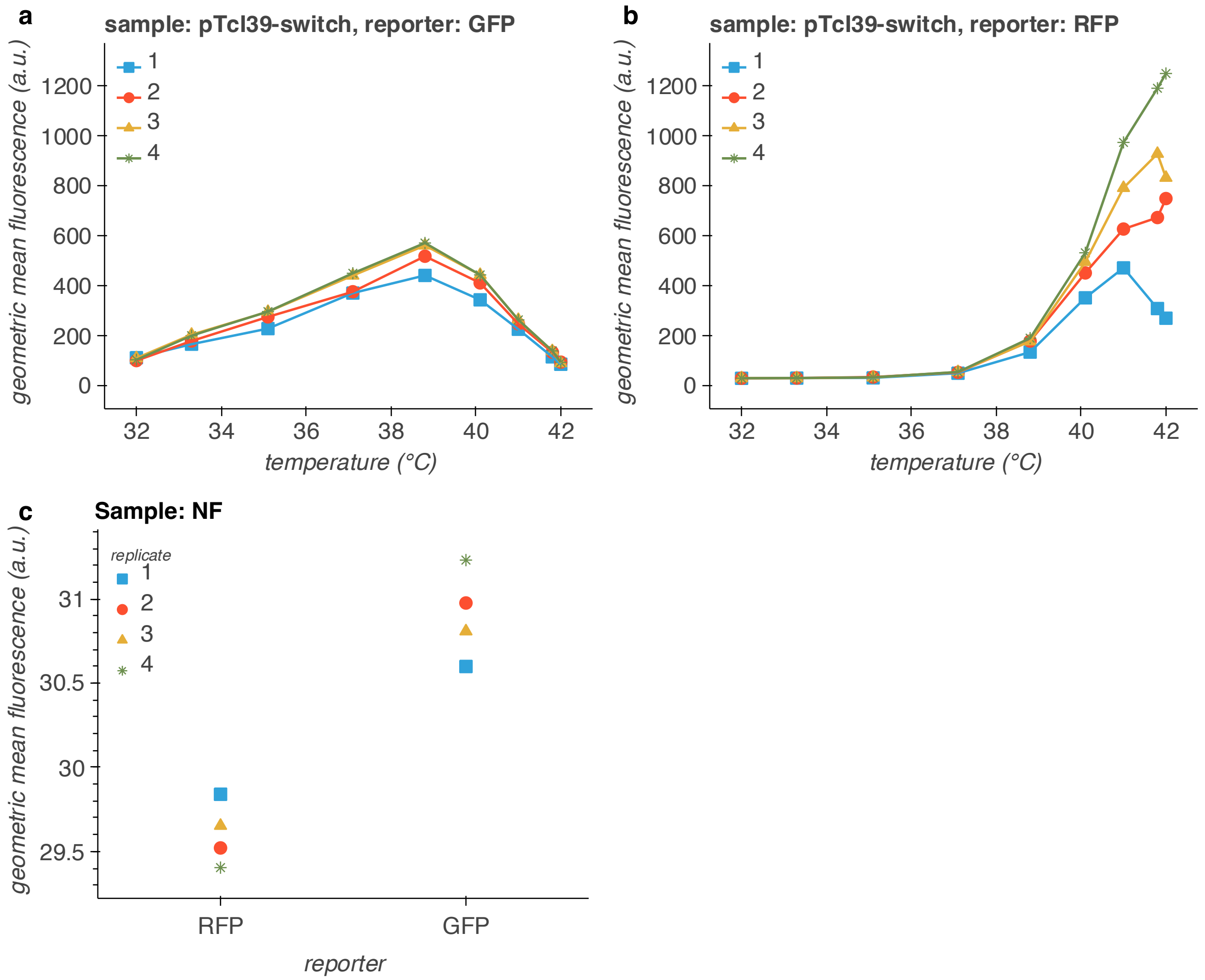

Supplementary figure S13. **(a, b)** Geometric mean of fluorescence in GFP channel **(a)** and RFP channel **(b)** for expression by TcI39 state switch construct, for each biological replicate. **(c)** Geometric mean of fluorescence in GFP channel and RFP channel for nonfluorescent controls grown at 37.1°C.

SUPPLEMENTARY REFERENCES

1. Ai, H., Olenych, S. G., Wong, P., Davidson, M. W. & Campbell, R. E. Hue-shifted monomeric variants of Clavularia cyan fluorescent protein: identification of the molecular determinants of color and applications in fluorescence imaging. *BMC Biol.* **6**, 13 (2008).
2. Piraner, D. I., Abedi, M. H., Moser, B. A., Lee-Gosselin, A. & Shapiro, M. G. Tunable thermal bioswitches for in vivo control of microbial therapeutics. *Nat. Chem. Biol.* **13**, 75–80 (2017).
3. McGinness, K. E., Baker, T. A. & Sauer, R. T. Engineering Controllable Protein Degradation. *Mol. Cell* **22**, 701–707 (2006).
4. Campbell, R. E. *et al.* A monomeric red fluorescent protein. *Proc. Natl. Acad. Sci.* **99**, 7877–7882 (2002).
5. EcoFlex: A Multifunctional MoClo Kit for E. coli Synthetic Biology | ACS Synthetic Biology. https://pubs.acs.org/doi/10.1021/acssynbio.6b00031.
6. Chen, Y.-J. *et al.* Characterization of 582 natural and synthetic terminators and quantification of their design constraints. *Nat. Methods* **10**, 659–664 (2013).
7. Der, B. S. *et al.* DNAplotlib: Programmable Visualization of Genetic Designs and Associated Data. *ACS Synth. Biol.* **6**, 1115–1119 (2017).
8. Teague, Brian. Cytoflow. https://github.com/cytoflow/cytoflow/.
9. Habib, E. A. E. Geometric mean for negative and zero values. *Int. J. Res. Rev. Appl. Sci.* **11**, 14 (2012).
10. Parks, D. R., Roederer, M. & Moore, W. A. A new "Logicle" display method avoids deceptive effects of logarithmic scaling for low signals and compensated data. *Cytometry A* **69A**, 541–551 (2006).
11. Moore, W. A. & Parks, D. R. Update for the logicle data scale including operational code implementations. *Cytometry A* **81A**, 273–277 (2012).
12. Teague, Brian. HOWTO: Use the logicle scale in other matplotlib plots. https://cytoflow.readthedocs.io/en/stable/dev_manual/howto/logicle.html.
13. Schneider, C. A., Rasband, W. S. & Eliceiri, K. W. NIH Image to ImageJ: 25 years of image analysis. *Nat. Methods* **9**, 671–675 (2012).
